# Comparison of Liquid and Solid-State Fermentation Processes for the Production of Enzymes and Beta-Glucan from Hulled Barley

**DOI:** 10.4014/jmb.2111.11002

**Published:** 2021-12-15

**Authors:** Se Yeon Lee, Chae Hun Ra

**Affiliations:** Department of Food Science and Biotechnology, College of Engineering, Global K-Food Research Center, Hankyong National University, Anseong-Si 17579, Republic of Korea

**Keywords:** Hulled barley, enzyme production, β-glucan, ergosterol, solid-state fermentation

## Abstract

Solid-state fermentation using hulled barley was carried out to produce enzymes and β-glucan. The one-factor-at-a-time experiments were carried out to determine the optimal composition of the basal medium. The modified synthetic medium composition in liquid-state fermentation was determined to be 70 g/l hulled barley, 0 g/l rice bran, 5 g/l soytone, and 6 g/l ascorbic acid. Optimal pretreatment conditions of hulled barley by solid-state fermentation were evaluated in terms of maximum production of fungal biomass, amylase, protease, and β-glucan, which were 1.26 mg/g, 31310.34 U/g, 2614.95 U/g, and 14.6% (w/w), respectively, at 60 min of pretreatment condition. Thus, the solid-state fermentation process was found to enhance the overall fermentation yields of hulled barley to produce high amounts of enzymes and β-glucan.

## Introduction

Barley is an ancient and important functional crop with soluble/insoluble dietary fibers and various active ingredients, such as fermentable sugars, amino acids, and vitamins [1]. However, the high cellulose content and the special protein composition of barley causes a certain palatability problem, whether it is used to produce flour or other products. Therefore, almost 80–90% of barley is used in animal feed and preparation of malt, and only a small part is used directly for human consumption [2]. Furthermore, there are no conclusive studies on the enzyme production profile of barley through solid-state fermentation (SSF) and other biochemical changes such as β-glucan content during fermentation. β-glucan, a well-known functional food ingredient derived from mushrooms, oats, and barely, possesses repeating structures with β-1,3 and β-1,4 bonds in their unbranched primary chains, and in fact, the effects of β-glucan on human immune and cancer cells have been reported [3]. Thus, we conducted studies on optimizing the production of enzymes and β-glucan using hulled barley. These study findings would help in better utilization of barley in food processing to save precious food resources and also improve the health of mankind.

The fermentation process can be performed under solid-state (SSF), submerged (SMF), or liquid conditions. Among these, SSF refers to the growth of microorganisms on an adequately moistened non-soluble medium in the absence or near absence of free-moving water. The advantages of SSF are high economic viability, use of cheap substrates, low production costs, high enzyme yields, and less energy consumption [4]. According to our previous study [5], SSF of brown rice showed significant potential for improving enzyme and β-glucan production. Thus, in this study, SSF using hulled barley was carried out to monitor its effects on enzyme and β-glucan production.

The filamentous fungus *Aspergillus oryzae* is an important strain in the traditional fermentation and food processing industries and is often used in the production of rice wine, soy sauce, and soybean paste [6]. Also, it has been used in the enzyme industry for the production of numerous native and heterologous enzymes (*e.g.*, amylase and protease). The advantages of *A. oryzae* include a strong synthesis ability, rapid growth, and ease of culture. Therefore, we employed the fungus *A. oryzae* NIBRFGC000501965 to optimize the production of enzymes and β-glucan using SSF.

The estimation of filamentous fungi enzyme activity or biomass is difficult due to hyphal tip growth. To solve these problems, ergosterol was used as a fungal biomarker for cell growth in SSF. The membrane lipid ergosterol is almost exclusively in fungi, and is frequently used by environmental microbiologists as an indicator of living fungal biomass [7]. Ergosterol has both free and esterified forms in fungi. The free form is located in cell membranes while the esters are found in cytosolic lipid particles [8]. According to the procedures described by Gessner *et al*. [9] and Klamer *et al*. [10], a strong correlation has been found between ergosterol, considered to be the most important sterol of fungi, and fungal biomass.

To derive the optimal parameters for improved enzyme and β-glucan production, in this study, the culture medium composition - particularly the hulled barley, nitrogen source, rice bran, and ascorbic acid concentrations were carefully evaluated to investigate the enzyme and β-glucan production capacity through SSF in 500 ml stainless steel rectangular trays.

## Materials and Methods

### Microbial Strains and Culture Medium

*Aspergillus oryzae* NIBRFGC000501965 was obtained from the National Institute of Biological Resources (NIBR, Korea). The culture was carried out as described in our previous reports [5]. Briefly, the spore suspension (1×10^8^ spores/ml) was inoculated in a 250-ml Erlenmeyer flask containing 100 ml of the modified synthetic medium comprising 70 g/l hulled barley powder, 5 g/l soytone, and 6 g/l ascorbic acid (Vitamin C). Ascorbic acid was added to the modified synthetic medium after sterile filtration (filter pore size of 0.22 μm). Nutrient supplements of 5 g/l K_2_HPO_4_ and 0.25 g/l MgSO_4_ were added to the modified synthetic medium and mixed together before inoculation. The culture was incubated for 48 h at 30°C and 150 rpm on a rotary shaker and then used as a seed culture.

### Effects of Synthetic Medium Condition

The effects of basal synthetic medium conditions were determined using one-factor-at-a-time (OFAT) experimental method, wherein only one factor is changed at one time while the other factors are kept fixed [11]. The basal synthetic medium was prepared with the following variable compositions: hulled barley concentration in the range of 10–90 g/l; rice bran concentration, 0–90 g/l; nitrogen source concentration, 5–40 g/l; and ascorbic acid concentration, 0–10 g/l. Various concentrations of hulled barley were individually prepared using 250-ml Erlenmeyer flasks, each containing 100 ml of the basal synthetic medium with 10 g/l yeast extract, 50 g/l rice bran, and 1 g/l ascorbic acid. After fixation of the hulled barley concentration, several factors influencing the basal synthetic medium composition were determined for optimal fungal biomass and enzyme production, including rice bran, nitrogen source, and ascorbic acid concentrations. Thereafter, fungal biomass and enzyme production was evaluated through liquid-state fermentation (LSF) of hulled barley by altering various synthetic medium conditions.

### Solid-State Fermentation (SSF)

100 g of hulled barley (Korea) was soaked with 150 ml water for 48 h in 500 ml rectangular trays (CY-1070; 260 × 170 × 50 mm, Chunyangsa Co., Ltd.,) and then the trays were closed with aluminum foil, autoclaved at 121°C for 60 min, and cooled to room temperature. Under aseptic conditions, nutrient supplements of fresh modified synthetic medium (5 ml) were added to the rectangular trays. Then, seed culture (10 ml) of *A. oryzae* NIBRFGC000501965 was inoculated onto the surface of autoclaved hulled barley and carefully mixed together. SSF was carried out at 30°C and 60% Rh (relative humidity at 20°C) using a Constant Temp & Humid Chamber (DS-150 TM; Daewon Science, Bucheon-si, Republic of Korea). Samples were collected periodically, and lyophilized using a freeze dryer (BFD85-F8; IlShinBioBase Co. Ltd., Korea), and stored at 20°C prior to the measurements of the amylase, protease, ergosterol, and β-glucan contents.

### Analysis of Fungal Biomass

The fungal growth during SSF was determined by the ergosterol analysis method proposed by Beni *et al*. [12]. Briefly, 0.5 g of the samples were placed into 15 ml conical tubes, and 10 ml of 0.07 M potassium hydroxide in methanol was added to each. Then, the tubes were placed in a shaking incubator (30°C, 150 ×*g*, 1 h) and extraction was carried out through ultrasonic shaking for 10 min. The supernatant from each extracting tube was harvested by centrifugation (994 ×*g*, 5 min) and was transferred to a new tube. To prevent ionization of ergosterol, 200 μl of 0.1 M HCl was added to each supernatant solution. The supernatant solution was then passed through the C-18 SPE column using Visiprep SPE Vacuum Manifold (Merck KGaA, Germany). The ergosterols retained on the C-18 SPE column were eluted with 2 ml of isobutanol. The concentration of ergosterol was determined via high-performance liquid chromatography (HPLC, Agilent 1200 Series; Agilent Technologies, USA) using a SUPERSIL ODS I-C18 column (250 × 4.6 mm). The UV detection was based on the photodiode array and was performed at a wavelength of 282 nm. The mobile phase consisted of 100% methanol at a flow rate of 1.0 ml/min and 30°C. The identification of each peak was based on a comparison of the dilutions of the standard solutions.

### Enzyme Assays

The activities of amylase and protease were determined according to the procedures described in previous reports [5, 13]. Amylase activity was carried out by measuring the reducing sugars released from soluble starch. One unit of amylase was defined as the amount of enzyme needed to liberate 1 μmol of glucose per min from starch under the below specified assay conditions. 1.0 ml of the sample (1 mg/ml) was mixed with 1.0 ml of starch solution (0.1 µg/ml), and the mixture was incubated at 37°C for 15 min in a water bath. The reaction was stopped by adding 2.0 ml of dinitrosalicylic acid (DNS) reagent and boiling for 5 min in a boiling water bath. The solution was cooled to room temperature and the absorbance was measured at 540 nm against a blank.

One unit of protease activity was defined as the quantity of enzyme required to release 1 μmol tyrosine per min from the substrate at 37°C. 1.0 ml of the solution of a protein substrate (0.1 mg casein/ml of 50 mM sodium phosphate buffer, pH 7.6) was mixed with 1 ml of the sample (50 µg/ml). This mixture was incubated at 37°C for 10 min. The protease activity was then quenched by adding 2 ml of 0.4 M trichloroacetic acid. A 1 ml aliquot of this supernatant was mixed with 5 ml of 0.4 M sodium carbonate buffer (pH 7.5) and 1 ml of diluted Folin reagent. After 30 min, the absorbance was measured at 660 nm. The blank was prepared exactly as above, but 2 ml of distilled water was used instead of the sample supernatant (1 ml) and the protein substrate (1 ml).

### Analysis of β-Glucan

The amount of β-glucan was analyzed using a mixed-linkage β-glucan assay kit supplied by Megazyme International. The activity of β-glucan was determined in accordance with the procedures described by Yoo *et al*.[14]. The β-glucan concentration was calculated using Eq. (1) as follows:

$$\text{β-glucan (\%,x/x) =ΔA×}\frac{\text{F}}{\text{W}}\text{×FV×0.9}$$ (1)

ΔA is the absorbance after applying β-glucosidase treatment to the reaction solution minus the absorbance of the reaction blank, F is a factor for converting absorbance values to the amount of glucose (μg), W is the weight of the extract analyzed (mg), and FV is the final volume (*i.e.*, 9.4 ml for rice flour). All analyses were conducted in triplicate, and the data were expressed as means ± standard deviation (SD).

## Results and Discussion

### Profiles of Liquid-State Fermentation (LSF)

**Fig. 1:**
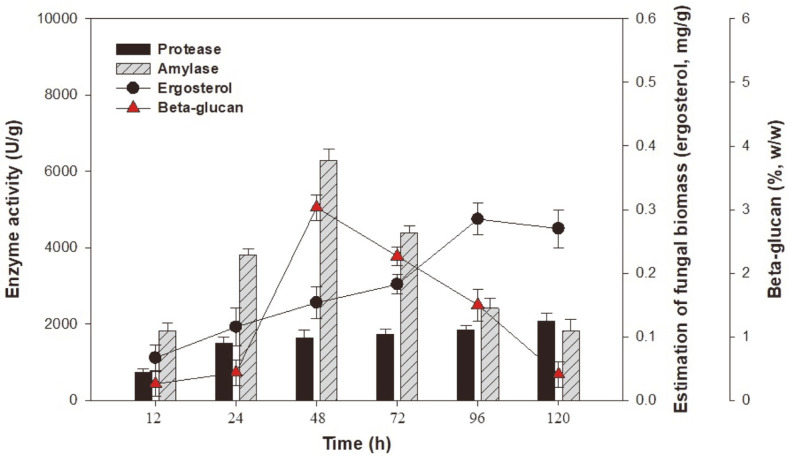
Fungal biomass and enzyme production as a result of LSF with synthetic medium. The initial pH was 5.5, the temperature was 30°C, and LSF was conducted for 120 h. The initial basal synthetic medium composition was 50 g/l hulled barley, 50 g/l rice bran, 10 g/l yeast extract, 1 g/l ascorbic acid, and nutrient supplements.

Simple LSF profiles were grown at 30°C, with 150 rpm for 120 h in 250-ml Erlenmeyer flasks containing 100 ml of the basal synthetic medium with 50 g/l of hulled barley, 10 g/l yeast extract, 50 g/l rice bran, and 1 g/l ascorbic acid. As shown in Fig. 1, LSF using the basal synthetic medium achieved the best culture time to produce high amounts of enzyme and beta-glucan. The growth of *A. oryzae* NIBRFGC000501965 reached the stationary phase at 96 h and led to 0.28 mg/g of the ergosterol concentration, considered as fungal biomass. Then, the productions of amylase and protease increased progressively with time. The highest enzyme and β-glucan yields were 6,294.82 U/g for amylase at 48 h, 2,075.72 U/g for protease at 120 h, and 3.03% (w/w) for β-glucan at 48 h. However, reduction in amylase and β-glucan production was observed at 48 to 120 h of the LSF. The initial pH of the medium (5.5) was decreased to 4.2 during fermentation (data not shown). This indicates that the pH of the synthetic medium is an important parameter for the enzyme and β-glucan production. According to Ellaiah *et al*.[15], glucoamylase production under SSF strongly depends on the extracellular pH, which affects several metabolic and enzymatic processes, thereby influencing the cell growth and metabolite production. Also, a lower or higher pH will affect the stability of extracellular enzymes and cause rapid denaturation [16]. Furthermore, some studies report that the optimal pH range for β-glucan production by bacteria is 5.5–7.0 [17]. Therefore, LSF of 48 h was selected as the optimal culture duration for enzyme and β-glucan production in this study.

### Effects of Hulled Barley and Rice Bran for Fungal Biomass and Enzyme Production

**Fig. 2:**
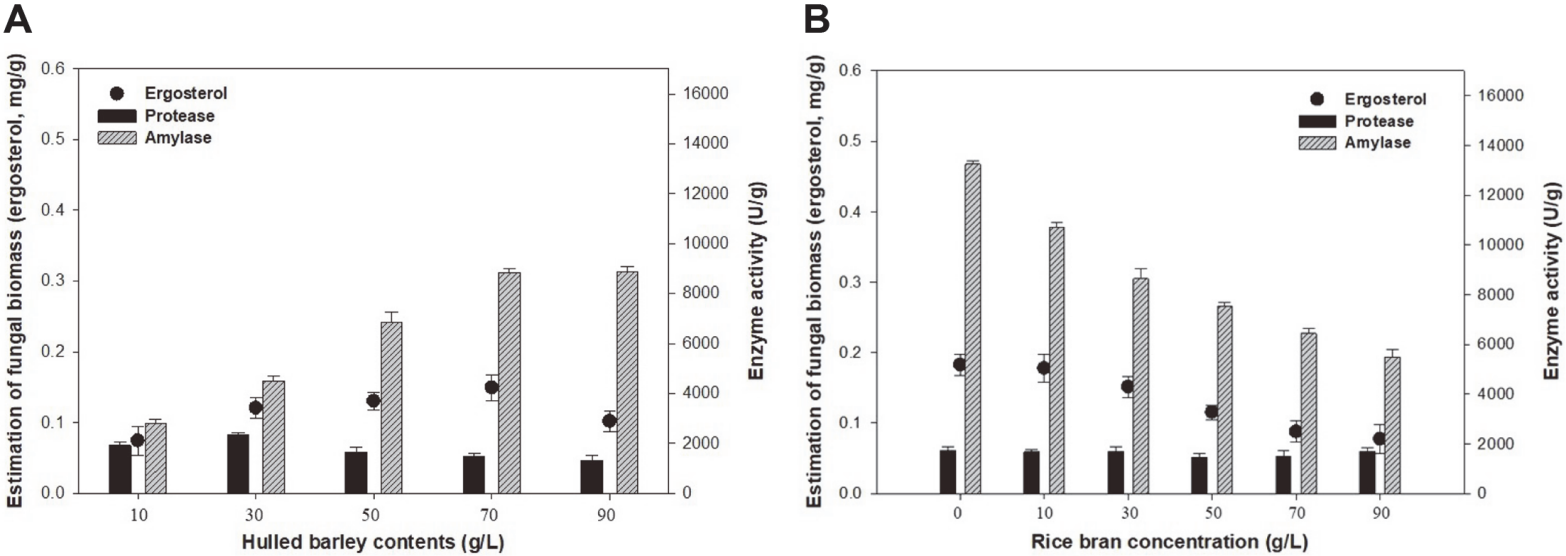
Effects of hulled barley (**A**) and rice bran concentration (**B**) on fungal biomass and enzyme production. The initial pH was 5.5, the temperature was 30°C, and LSF was conducted for 48 h. The modified synthetic medium composition was 70 g/l hulled barley, 0 g/l rice bran, 10 g/l yeast extract, 1 g/l ascorbic acid, and nutrient supplements.

Evaluation of fungal biomass and enzyme production in LSF subcultures was carried out by changing the synthetic medium compositions. The effects of hulled barley (Fig. 2A) and rice bran concentrations (Fig. 2B) in LSF were evaluated at 30°C and 150 rpm for 48 h using *A. oryzae* NIBRFGC000501965.

Fig. 2A shows the fungal biomass and enzyme production under different hulled barley concentrations. It was observed that the optimal concentration of hulled barley was 70 g/l; increasing the hulled barley concentration to 90 g/l had no significant effects on fungal biomass and enzyme production. Moreover, it was apparent that the concentration of protease tended to decrease with increasing hulled barley concentration. The highest fungal biomass, amylase, and protease concentrations obtained were 0.15 mg/g, 8,832.20 U/g, and 2,324.68 U/g, respectively. Based on the results shown in Fig. 2A, 70 g/l was chosen as the suitable hulled barley concentration in LSF subculture.

As shown in Fig. 2B, after the hulled barley concentration had been fixed at 70 g/l, the basal synthetic medium was supplemented with various concentrations of rice bran. Our results revealed that the best rice bran concentration was 0 g/l and the optimal fungal biomass, amylase, and protease yields were 0.18 mg/g, 13,244.82 U/g, and 1,724.13 U/g, respectively. Notably, a further increase in rice bran concentration to 90 g/l had no significant effect on the fungal biomass and enzyme production. The inhibitory effects were probably caused by the high amount of fibrous hull in hulled barley. These results indicate that the basal synthetic medium with hulled barley may validate the lack of need for rice bran. Thus, 0 g/l of the rice bran was selected as a suitable synthetic medium condition.

On the other hand, several studies have explored the effects of rice bran fermentation on its functional properties. After fermentation, an increase in nutrient availability, biosurfactant content, and mono- and polyunsaturated fatty acid content was observed [18-20]. Therefore, rice bran can be employed as a synthetic medium composition for microorganisms and fungus in SSF, allowing the production of natural bioactive compounds, enzymes, and other products such as feed additives for animals.

### Effects of Nitrogen Sources and Ascorbic Acid for Fungal Biomass and Enzyme Production

**Fig. 3:**
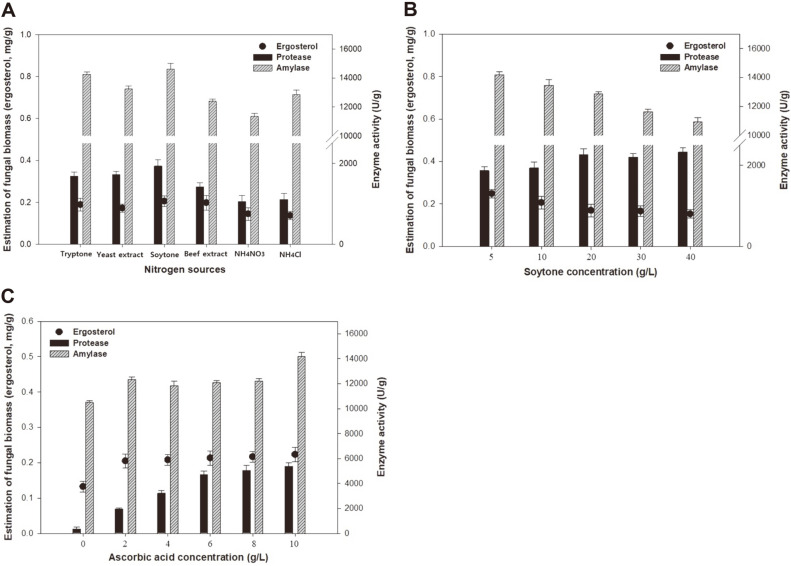
Effects of the nitrogen source (**A**), soytone concentration (**B**), and ascorbic acid concentration (**C**) on the fungal biomass and enzyme production. The initial pH was 5.5, the temperature was 30°C, and LSF was conducted for 48 h. The modified synthetic medium composition was 70 g/l hulled barley, 5 g/l soytone, 6 g/l ascorbic acid, and nutrient supplements.

LSF subculture was carried out for 48 h to determine the best nitrogen source and ascorbic acid concentrations as shown in Fig. 3. As shown in Fig. 3A, various nitrogen sources such as 10 g/l of yeast extract, tryptone, beef extract, soytone, NH_4_Cl, and NH_4_NO_3_ were evaluated for their effects on fungal biomass and enzyme production using a basal synthetic medium.

Among the various nitrogen sources, soytone produced the highest fungal biomass, amylase, and protease yields: 0.21 mg/g, 14,619.78 U/g, and 1,934.85 U/g, respectively. Thus, soytone (enzymatic digest of soybean meal) was chosen as the ideal nitrogen source for fungal biomass and enzyme production. Similar results were obtained for *A. oryzae* CBS 819.72 [21]. It was also observed that KH_2_PO_4_, urea, glycerol, (NH_4_)_2_SO_4_, CoCl_2_, casein hydrolysate, soybean meal hydrolysate, MgSO_4_ were selected based on their positive influence on enzyme formation.

As shown in Fig. 3B, protease production of *A. oryzae* NIBRFGC000501965 increased as the soytone concentration increased to 40 g/l. However, fungal biomass and amylase production decreased with an increase in the soytone concentration from 10 to 40 g/l. The highest productions of fungal biomass of 0.25 mg/g, amylase of 14,193.10 U/g, and protease of 1,867.37 U/g, respectively, were obtained with the soytone concentration at 5 g/l. Thus, 5 g/l soytone was selected as the preferred concentration for subsequent experiments.

As shown in Fig. 3C, the effect of ascorbic acid concentration on the fungal biomass and enzyme production was assessed with concentrations of 0–10.0 g/l. The suitable fungal biomass, amylase, and protease yields of 0.22 mg/g, 12,078.23 U/g, and 4,695.20 U/g respectively, were obtained at an ascorbic acid concentration of 6.0 g/l for 48 h. With an increase in the ascorbic acid concentration above 2.0 g/l, LSF subculture had no significant effect on the fungal biomass and amylase production. However, the enzymatic activity of protease increased when the ascorbic acid concentration was increased from 2.0 to 6.0 g/l. Similar results were obtained for *A. oryzae* in rice *koji*, which suggested that vitamins were related to the synthesis of fatty acids and that vitamins participated in the energy supply of the cells [22]. In addition, enzymes involved in fermentation require vitamins as cofactors. Therefore, 6.0 g/l ascorbic acid concentration was selected for efficient enzyme production.

### Solid-State Fermentation (SSF) of Hulled Barley

**Fig. 4:**
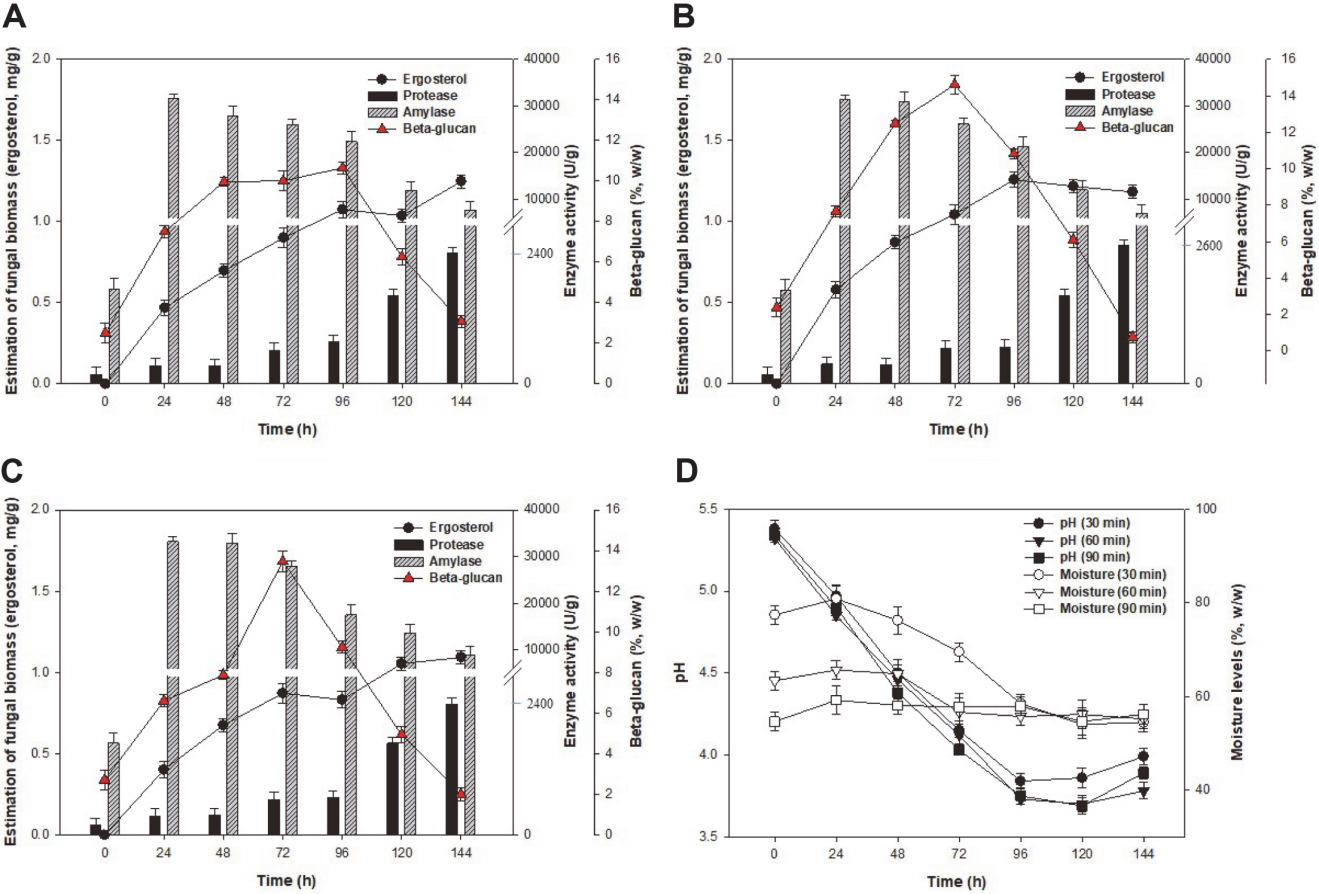
Effects of various pretreatment conditions on the production of fungal biomass, enzymes, and β-glucan; (**A**) 30 min of pretreatment, (**B**) 60 min of pretreatment, (**C**) 90 min of pretreatment, and (**D**) levels of pH and moisture. SSF was carried out at 30°C and 60% Rh (relative humidity at 20°C) for 144 h.

SSF of hulled barley by *A. oryzae* NIBRFGC000501965 with different pretreatment conditions were compared in terms of fungal biomass, enzyme, and β-glucan production. As shown in Fig. 4, the pretreatment conditions of 30, 60, 120 min were evaluated using 500 ml rectangular trays with a working volume of 150 ml.

As shown in Fig. 4A, the growth of fungal biomass with pretreatment of 30 min reached the stationary phase at 96 h and produced a fungal biomass of 1.07 mg/g. Notably, the protease production increased during fermentation, whereas amylase production decreased from 24 to 144 h. The highest enzyme activities of SSF were 31,655.17 U/g for amylase production at 24 h and 2,474.60 U/g for protease production at 144 h, respectively. Furthermore, β-glucan production increased to 10.64% (w/w) after 96 h of fermentation, and then decreased gradually from 10.64 to 3.06% (w/w) at the end of fermentation. When the moisture level and the pH of broth were decreased from 77% to 54% and 5.3 to 3.8 respectively at 120 h (Fig. 4D), protease production increased, while the production of fungal biomass, amylase, and β-glucan decreased. These results indicate that initial moisture levels and the final pH of broth are critical for the syntheses of fungal biomass, enzymes, and β-glucan production. Similar results were obtained for SSF of *Streptomyces* sp. strain MAR01 [23]. The authors reported that the high moisture content may reduce the porosity of the wheat bran, and thus limit oxygen and mass transfer. In contrast, the low moisture content would inhibit microbial growth and enzyme production, and limit nutritional transfer [24]. Therefore, the solid substrate should possess the appropriate level of available moisture to support microbial growth and metabolism.

Fig. 4B shows the impact of pretreatment condition of 60 min on the fungal biomass, amylase, protease, and β-glucan production assessed using the SSF process. Each of the products was rapidly fermented and maximum concentration of the fungal biomass, amylase, protease, and β-glucan were 1.26 mg/g at 96 h, 31,310.34 U/g at 24 h, 2,614.95 U/g at 144 h, and 14.6% (w/w) at 72 h, respectively. In addition, the pH decreased to 3.7, and the moisture content decreased from 63% to 55% at 120 h of fermentation (Fig. 4D). These results showed that 60 min of pretreatment in the SSF process had a positive effect on the yields of fungal biomass, enzymes, and β-glucan compared to those obtained with 30 min of pretreatment condition (Fig. 4A). This may be due to the reason that proper pretreatment time plays a key role in the moisture content during SSF, which increases the hydration homogeneity, degree of gelatinization, percentage of broken kernels, and degree of starch leaching, resulting in the easy penetration of mycelia into the substrate [25]. Thus, 60 min of pretreatment was chosen as the best pretreatment condition in this study.

Fig. 4C shows the production of fungal biomass, enzymes, and β-glucan with 90 min of pretreatment on hulled barley in SSF. The growth of fungal biomass, 0.83 mg/g at 96 h, was slightly lower than that at 30 min (Fig. 4A) or 60 min (Fig. 4B) of pretreatment conditions. The maximum concentrations of fungal biomass, amylase, protease, and β-glucan were 1.09 mg/g at 144 h, 33,206.89 U/g at 24 h, 2,474.60 U/g at 144 h, and 13.4% (w/w) at 72 h, respectively. Furthermore, the pH decreased to 3.7 at 96 h, and the moisture level remained the same at 54% at 120 h of fermentation (Fig. 4D). With regards to the moisture content, SSF showed similar trends as the others, even at 90 min of pretreatment condition. These results indicate that increase in the pretreatment time did not lead to significant increase in product yields due to the low moisture content and inferior substrate texture [26]. Indeed, the texture of hulled barley was harder and stickier than that at other pretreatment conditions.

Figs. 4A-4C and 4D show that the metabolic activities of *A. oryzae* NIBRFGC000501965 were sensitive to pH changes, which had a negative effect on the amylase production. Similar results were obtained previously for the solid fermentation of wheat bran for hydrolytic enzyme production, which showed maximum production of pectinase, xylanase and α-amylase by *Bacillus megatherium* at pH 7, 6 and 5, respectively [27]. To overcome the problems caused by the instability of enzymes during SSF, buffer system techniques have been developed for use in the nutrient medium [28]. Therefore, in SSF, the moisture content and pH of the fermentation medium are important factors that influence the growth and product yield of microorganisms.

Furthermore, a decrease in β-glucan production was observed during SSF (Figs. 4A-4C). A similar phenomenon was observed for lactic acid bacteria (LAB) fermentation using native oat and barley fiber concentrates; the amount of soluble β-glucan in the oat and barley fiber concentrates decreased significantly during fermentation by LAB [29]. The mechanism behind this is not clear, but it is most likely an enzymatic breakdown similar to that of cellulose degradation [30]. Based on these results, SSF process was shown to be the most effective production technique for fungal biomass, enzymes, and β-glucan from hulled barley.

*A. oryzae* NIBRFGC000501965 produced greater fungal biomass and higher yields of enzymes and β-glucan under SSF than LSF. OFAT experiments were performed with suitable synthetic medium compositions under LSF process. SSF of hulled barley with 60 min of pretreatment condition was concluded to produce higher fungal biomass and enzyme and β-glucan concentrations than the other pretreatment conditions. Additionally, proper moisture levels and stability of pH are important factors that influence the growth and product yield of microorganisms. Therefore, the various profiles of SSF are thought to provide a viable base for optimal enzyme and β-glucan production.
